# Effects of Tourism on the Habitat Use by a Threatened Large Rodent at a World Heritage Site

**DOI:** 10.3390/ani11082278

**Published:** 2021-08-02

**Authors:** Veronica A. Beninato, Carlos E. Borghi, Natalia Andino, Mauricio A. Pérez, Stella M. Giannoni

**Affiliations:** 1 Centro de Investigación de la Geósfera y la Biósfera, Facultad de Ciencias Exactas, Físicas y Naturales, Universidad Nacional de San Juan-Consejo Nacional de Investigaciones Científicas y Técnicas (CONICET), Complejo Universitario Islas Malvinas, Ignacio de la Roza 590 (Oeste), Rivadavia, San Juan J5402DCS, Argentina; vbeninato@unsj-cuim.edu.ar (V.A.B.); nandino@unsj-cuim.edu.ar (N.A.); elcodorniz81@gmail.com (M.A.P.); 2 INTERBIODES, Departamento de Biología, Facultad de Ciencias Exactas, Físicas y Naturales, Universidad Nacional de San Juan, Complejo Universitario Islas Malvinas, Av. Ignacio de la Roza 590 (Oeste), Rivadavia, San Juan J5402DCS, Argentina; 3 Instituto y Museo de Ciencias Naturales, Facultad de Ciencias Exactas, Físicas y Naturales, Universidad Nacional de San Juan, España 400 (N), San Juan J5400DCS, Argentina

**Keywords:** habitat use, endemic species, tourism conflict, conservation, World Heritage sites

## Abstract

**Simple Summary:**

The mara is a large endemic rodent, which major threats are habitat loss, hunting, and overgrazing. Maras live in arid and semiarid areas of Argentina. We studied the influence of environment variables and tourist activity on mara’s habitat use. We used different ecological approaches, from plant communities to floristic composition, in order to know at which level we can better detect the tourism effects on mara’s habitat use. We counted feces of maras as a habitat use index and recorded environmental variables along 80 samples in two plant communities, near and away-from the tourist circuit. To evaluate habitat use, we made statistical models using plant communities, plant strata, cover of trees, shrubs, and grasses, and plant species abundance as explaining factors. We detected the tourism effects on habitat use utilizing cover of trees, shrubs, and grasses, and cover of more abundant plant species, but not plant communities and plant strata, as explicative factors. Maras also selected areas with low bare soil with few pebbles on it. We found complex interactions between abiotic, biotic, and anthropic variables, studying maras’ preferred places near tourism activities, which they probably perceive as safer from predators.

**Abstract:**

The mara is a large endemic rodent, which presents a marked decline in its populations, mainly because of habitat loss, hunting, and overgrazing. The Ischigualasto Provincial Park is a hyper-arid protected area at the Monte Desert of Argentina with an overall low plant cover. Our objective was to determine the influence of environmental variables and tourist activities on mara’s habitat use. We used different biological levels to explain it, from plant community to floristic composition, in order to know at which level we can better detect the effects of tourist activities. We registered fresh feces and habitat variables along 80 transects in two communities, near and far away from the tourist circuit. To evaluate habitat use, we fitted models at different biological levels: plant community, plant strata, plant biological forms, and floristic composition. At the community and plant strata levels, we could not detect any tourism effects on habitat use. However, we detected effects of tourist activities on mara’s habitat use at the plant strata and floristic composition levels. Maras also selected areas with a low proportion of both bare soil and pebbles cover. We found complex interactions between abiotic, biotic variables and tourism, studying mara’s places near tourism activities, probably because they perceive those places as predator-safe areas.

## 1. Introduction

Knowledge of the land cover types in which a species occurs can provide valuable insights into its biology, including aspects of its ecology, behavior, and conservation [1]. This information can be applied to improve management actions for threatened and restricted distribution species, mitigating human−wildlife conflicts [2]. The degree of flexibility in the use of habitat is likely to be important in determining species vulnerability, especially in anthropogenic environments where certain land covers may be selectively retained and others lost. Moreover, a rigid requirement for multiple land covers for a species would increase the risk of it becoming locally extinct [1].

The mara or Patagonian hare (*Dolichotis patagonum*, Order Rodentia, Suborder Hystricomorpha, Family Caviidae) is endemic to Argentina and the second largest rodent of the world (body mass between 7 and 9 kg) [3]. It is a social monogamous species, with a flexible reproductive behavior [4,5]. Female maras achieve sexual maturity at 8 months of age [6], and have between one to two litters per year, with a litter size ranging from one to three pups [5]. Maras are diurnal [7], have a cursorial locomotion style, and are the fastest known rodent [8]. This species is considered convergent with small artiodactyls, showing an evolutionary parallelism to small sized artiodactyls, as evidenced by the anatomy and biomechanics of their long bones, related to its anatomical traits associated with its fast locomotion [8]. Maras are herbivores that feed mainly on grasses [7,9], but they also eat forbs, shrubs, and cacti when grasses are scarce [10]. Its conservation status at is “near threatened” the international level [11] and “vulnerable” at the country level [3], with an estimated decreasing population trend of >30% in the last 10 years [3,11]. This species had a wide distribution in Argentina, occurring from north central Argentina to the south of it, Santa Cruz province [7,12], in the ecoregions of Dry Chaco, Espinal, Patagonian steppe, and Monte Desert [13]. Across its geographical range, mara is restricted to arid lands, where it selects sparsely vegetated areas with a high proportion of bare soil [14,15,16]. It was also suggested that the mara could benefit from human-made environments, where it also increases its predator detection efficiency [14].

Some studies found a potentially favorable effects of certain human activities on the population of mara (e.g. fire, logging, cropped, dirt road, and trail), because these generate open environments with high cover of bare soil [14,16,17]. However, humans are related to the main processes threatening the populations of maras, such as the loss of habitat, overhunting, and competition with alien species that have similar diets, such as domestic herbivores as sheep (*Ovis orientalis aries*) or introduced wild European hares (*Lepus europaeus*) [3,7,10,16]. In every distribution area, this species is quickly disappearing, and protected areas remain as isolated ecological islands for it. Nevertheless, it does not respond in the same way in all geographic areas, and there may be context-dependence in its response to protected areas [17]. However, some protected areas have an intense tourist activities, and until now, we do not know the effects of tourism on mara habitat use.

Wildlife tourism is a non-consumptive activity, which has been assumed to have no long-term impact on the ecological processes of an area [18]. However, tourism can produce a wide range of negative impacts that occur at multiple levels, from individuals to communities [19,20], when the ecological tourist carrying capacity is exceeded [21]. Humans have negative effects on, among others, vigilance rates, vigilance duration, and feeding behavior of species [20]. At a community level, tourist activities cause, for instance, a decrease in avian species richness and diversity [22]. Humans can also cause disturbances that trigger anti-predator responses similar to real predation risk, e.g., lower flight initiation distance and greater distance moved than non-hunted populations [23,24]. These disturbances may be greater in protected areas where it is very difficult to completely eradicate poaching. Nevertheless, tourism in protected areas still is an important tool for environmental conservation and for social and economic development [25].

In general, protected areas draw vast numbers of visitors, which leads to a potential conflict between conservation and tourism development [26]. This could be the case for many threatened species who live in protected areas of developing countries, which are under high and increasing tourist pressure. This type of conflict is becoming increasingly common at World Heritage sites, because the tourist activity in them is increasing [27], which poses a great challenge, namely, to achieve ecological sustainability of tourism at these sites. In those protected areas, it is necessary to know the habitat requirements of these species and how tourism affects them, to allow tourism below the tourism carrying capacity. This is the case of the mara at the Ischigualasto Provincial Park, a World Heritage site located at the Monte of Mountains and Basins ecoregion, where maras interact with an increasing tourist activity.

Within this framework, the objectives of this study were to determine the influence of the natural environment variables (biotic and abiotic) versus the anthropic variable, tourist activity, on maras in the Ischigualasto Provincial Park, one of the largest contiguous landscapes in the hyper-arid Monte desert of Argentina. We used different biological levels to explain the habitat use of maras, from plant community to floristic composition, in order to know at which level we can better detect the effects of tourist activities. Our hypothesis was that the current tourism intensity negatively affects the habitat use by mara. Therefore, we expected maras to minimize human encounters, using the areas where there was less tourist activities.

## 2. Materials and Methods

**Figure 1:**
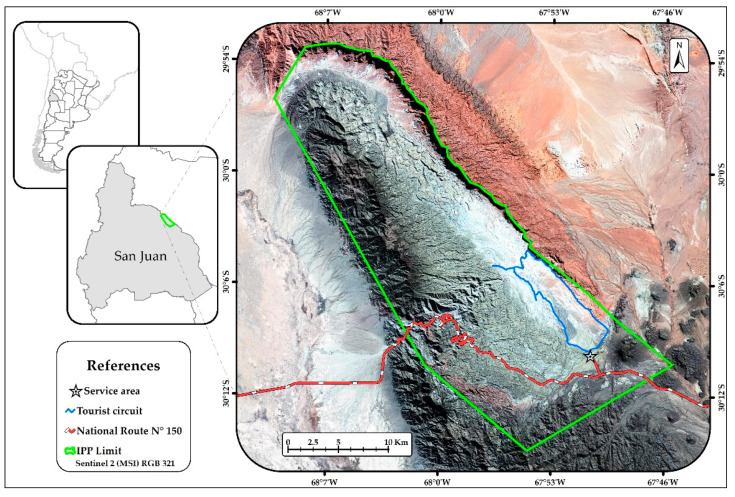
Geographic location of the Ischigualasto Provincial Park (IPP) in San Juan, Argentina. The tourist circuit is highlighted in blue.

The study was conducted in the Ischigualasto Provincial Park (30°05′S–67°55′W) in San Juan Province, Argentina (Figure 1). This protected area, together with the Talampaya National Park makes up the Ischigualasto–Talampaya World Heritage site (UNESCO). The number of tourists visiting the Ischigualasto Provincial Park has increased by almost 500% in the last 20 years, from just over 15,000 to almost 100,000 visitors a year [28,29]. The park extends over 62,916 ha at a mean altitude of 1300 m and Monte Desert biome dominates this hyper-arid system [30]. The overall plant cover is low (nearly 15%), and the vegetation consists of an open landscape, dominated by the shrubs species *Larrea* spp., *Plectrocarpa tetracantha*, *Atriplex* spp., and *Suaeda divaricata*, the trees *Prosopis chilensis*, *P. flexuosa*, and *Ramorinoa girolae*, the columnar cacti *Echinopsis terscheckii*, and a low and seasonal herbaceous layer [31]. These plant species are sorted in six plant communities [31]. The region has desert climate, with summer rains below 100 mm per year, absolute minimum temperatures of about −10 °C in winter and absolute maximum temperatures of around 45 °C in summer.

Mesquite woodland and saltbush were the communities we considered when analyzing the influence of the tourist activity on habitat maras use. These communities are crossed by a 40 km long tourist dirt road, which runs across nearly 30% of the total protected area [28]. Mesquite woodland is a gallery forest associated with seasonal rivers, dominated by *Prosopis chilensis,* with a mean tree cover reaching 11.97% ± 1.08%, and saltbush community is dominated by *Atriplex lampa* and *A. spegazzinii*, with a mean saltbush cover of 12.13% ± 0.95% [31].

**Figure 2:**
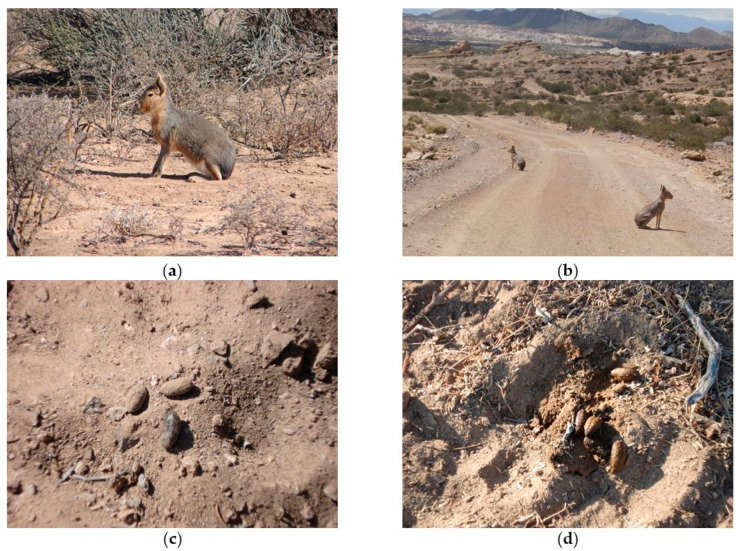
Photos of adults and feces of maras (*Dolichotis patagonum*) from the Ischigualasto Provincial Park, in San Juan, Argentina: (**a**) an adult; (**b**) a couple of maras sitting on the tourist dirt road; (**c**) feces detail; (**d**) a group of feces.

Mara occurs in low abundance and is elusive; therefore, we used fresh feces as a proxy of habitat use (Figure 2) [32]. Counting of feces has been broadly used in ecology as an indirect indicator of habitat use, diet, and spatial segregation in medium to large-sized herbivores [32,33,34]. Furthermore, some of us found that feces count and camera traps gave similar results about the space use of guanacos in the same protected area [35]. Moreover, feces of mara are impossible to confuse with feces of another animal that occurs in the study area, due to their characteristic shape and size (Figure 2c,d); this indirect sampling method has already been used to study this species [14,17].

We evaluated the habitat use of maras between August and November, in the two plant communities studied (mesquite woodland and saltbush), in two different tourist disturbance situations (areas with high and low tourism impact), using a factorial design. In Ischigualasto, tourists only enter in vehicles and circulate along the tourist circuit (dirt road), along which there are selected tourist stops, where visitors descend from their cars to observe the landscape. The human impact is restricted to the areas near the National Route 150 (used as an access road to the park), the service area of the park, and along the tourist circuit (Figure 1). We defined the areas near this tourist circuit (between 30 and 150 m) as “areas with high tourist impact”, and areas distant from the tourist circuit (between 1500 and 2000 m) as “areas with low tourism impact”, since large mammals can be affected by noise up to at least 1 km away [36,37]. We quantified fresh mara feces along 40 random 60 m long transects in each plant community (>200 m apart) between August 2009 and March 2010 (20 near the tourist circuit and 20 away from it, in each community). On each transect, we set up 10 subsamples (3 m × 3 m quadrats) separated by 3 m, where feces were recorded. For each transect, we also recorded the plant community type (mesquite woodland or saltbush). In 5 of the 10 quadrats (separated by 6 m), the percentage of cover of abiotic and biotic habitat variables was visually estimated and recorded (bare soil, pebbles, and cover of plant species). Plants were categorized by their height (four strata: <0.26 cm, 0.26–0.69 cm, 0.7–0.99, and >1 m), and in life-forms (four classes: forbs and grasses, shrubs, trees, and cacti). The percentual cover of each plant stratum and life-form was estimated as the median (5 subsamples) of the sum of the plants that belong to each category in each subsample. Even though we identified all plant species, in the analyses, we used only species having at least 1% cover in one of the treatments. Sampled units were transects, and each transect was sampled only once.

We used the generalized linear model (GLM) to model the data of mara’s habitat use. We used a multimodel inference approach to assess the relative effect of each predictor on the response variable. We constructed models using all combinations of predictors and computed the Akaike Information Criterion corrected for small samples (AICc) for each of the candidate models [38]. Akaike’s information criterion was calculated to evaluate the models that best fitted the data [38]. The difference between the lowest AICc value and the AICc from all the other models (ΔAICc) was calculated to rank the candidate models, and we selected the models with the lowest AICc value [38]. To build models, we used the frequency of mara feces as a response variable. We chose the median rather than the mean of frequency mara feces because the mean is a poor estimator of the central tendency for variables that are not normally distributed, such as the ones we sampled. As predictor variables, we used the plant community (two levels: mesquite woodland and saltbush), cover of bare soil and pebbles, cover of plant species, cover of plant strata and cover of a different biological form (explanatory continuous variable), and tourist activity (two levels: areas with high tourist impact and areas with low tourism impact). We applied negative binomial error structure because the data exhibited overdispersion (c > 1). For each biological level, we adjusted models with all possible combinations of predictor variables as well as interaction among them using the dredge function of MuMin library [39]. In all cases, we evaluated the relative importance (RI) of each predictor variable [38] and the percentage of the total variance explained for each model selected using library “rms ” for a binomial distribution of data [40]. All statistical analyses were performed using R Core Team (2019), version 3.6.1 [41].

## 3. Results

**Figure 3:**
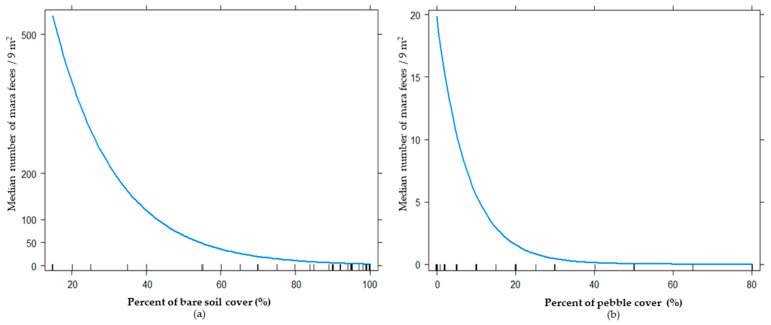
Relationship between habitat use of maras (*Dolichotis patagonum*) measured as median number of mara feces by quadrat (3 m × 3 m) and abiotic variables of models for plant communities and plant strata levels: (**a**) relationship between habitat use of maras and bare soil cover, (**b**) relationship between habitat use of maras and pebble cover.

**Figure 4:**
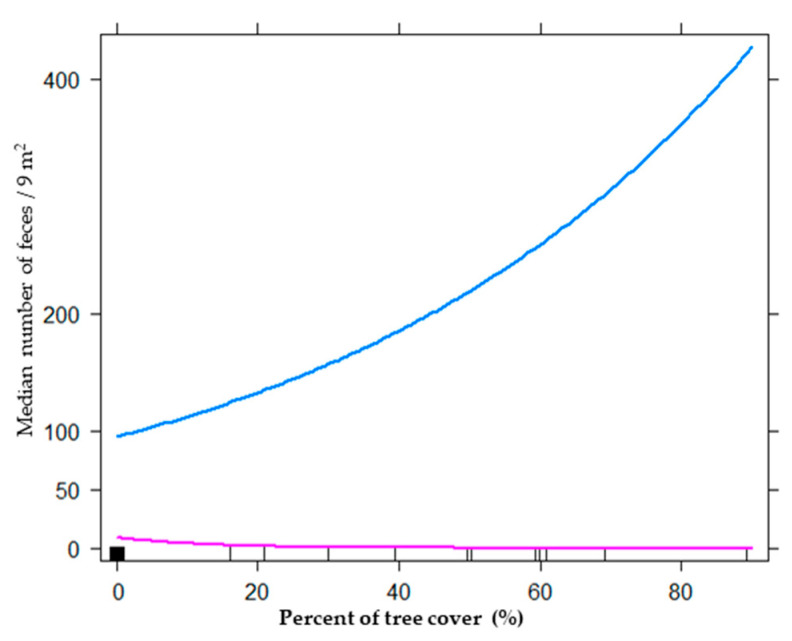
Interaction of tourism activity and tree cover with habitat use of maras (*Dolichotis patagonum*), measured as median number of mara feces by quadrat (3 m × 3 m), as a response variable, of models for biological plant forms and floristic composition levels: (a) blue line, near tourist circuit (high tourist activity), (b) pink line, distant tourist circuit (low tourist activity).

To assess the effects of tourism activities on habitat use of mara, we built models for four biological levels: plant community (35 models), plant strata (550 models), plant biological form (275 models), and floristic composition (440 models). The best model for the plant community level included the additive effect between pebble cover and bare soil (Table 1). The total deviance explained by the best model was 15.48%, and the relative importance (RI) of pebble cover and bare soil showed high values close to one (0.99 and 0.93, respectively). When we used biological variables at the plant strata level, the best model obtained was the same (Table 1). The estimate parameter analyses showed that with an increase in bare soil cover and pebble cover, the frequency of mara feces diminished (estimate: −0.06 and −0.12, respectively; Figure 2). For the biological forms level, the best model included tourist activity, pebble cover, bare soil, trees, and an interaction of tourist activity and pebble cover with tourist activity and trees (Table 1). All these variables explained 31.32% of the total deviance, and its RI was >0.80. When we used floristic cover as a predictor, we found an additive effect of tourist activity, *Prosopis*, pebble cover, bare soil, and an interaction of tourism activity, *Prosopis,* and tourist activity with pebble cover (Table 1; Figure 3). The bests models at the biological life-forms and floristic levels were the same, because the only trees recorded were *Prosopis* spp, and as the only biological form selected were trees, the data were the same. In both cases, the estimate parameters took values of 8.03 for an interaction of tree cover with high tourist activity, and −0.10 for tree cover with low tourist activity (Figure 4).

## 4. Discussion

Contrary to what we expected, the hypothesis that current tourism intensity negatively affected habitat use by mara was not validated by the results obtained in this study. Our results revealed that in the Ischigualasto Provincial Park, at the community and plant strata levels, the main variables that explained the habitat use of maras were pebble cover and bare soil. These models explained up to 15.5% of the deviance observed in mara habitat use. When we considered the physiognomy level, the cover of different plant forms as explicative biological variables, the best model included, in addition to the abiotic variables (pebble and bare soil cover), the tourist activity pressure (areas near the tourist circuit vs. areas far away from it), tree cover, and the interactions between tourist activity and pebble cover, and between tourist activity and tree cover. This model explains 31.2% of the observed deviance and was the best among the models developed for the four biological levels. Finally, when we included the floristic composition in the model, the best model also included pebble and bare soil cover as abiotic variables, tourist activity and *Prosopis* spp. cover as in the previous model, and the interactions between tourist activity and pebble cover and *Prosopis* spp. cover. These last interactions probably are associated with the predation risk perceived by maras in contrasting environments (*Prosopis* cover and high pebble cover), but further studies are needed to validate this interpretation. Nevertheless, the effects of tourist activities was the contrary to what we expected, because mara used more the areas near the tourist circuit.

Our results highlighted the value of abiotic variables in mara’s selection of environmental categories identified in previous studies on the mara [14,15,16,42]. Nevertheless, while previous studies have found that maras positively selected bare soil [16], we found that they were negatively associated with a high proportion of bare soil and pebble cover (Figure 3). Our results also emphasized the main importance of the biological level in order to best understand the habitat use of maras. Clearly, the plant cover of different biological forms is very important in the habitat selection process of the mara. Our results showed that mara selected trees and/or *Prosopis* sp.A positive association of the mara with trees or *Prosopis* sp. (mesquite woodland) was only recently reported in the semiarid reserve of Ñacuñan and adjacent rangelands [17].

In rangelands outside the protected area, these authors [17] found that maras were associated with low plant cover in *Prosopis* woodland (≈40%). Our study area had a very low vegetation cover (<15%), but it was inside a protected hyper arid area, which resembled the degraded *Prosopis* woodland in a semiarid environment [14,16,17]. Furthermore, this association with trees is logical, given the importance of *Prosopis* fruits in the maras’ diet [10].

Although both studied plant communities at our site offer open environments to the mara, the resources they provide are different. Mesquite woodland provides food, such as *Prosopis* spp. fruits and leaves, which are heavily consumed [10]. It also provides shielding from intense solar radiation. Saltbush community is dominated by shrubs of *Atriplex* spp., which is the most selected food of the mara [10]; moreover, the substrate where they grow is suitable for building their burrows. Nevertheless, at our study sites, even in areas with the highest vegetation cover, early detection of predators, an important feature of mara habitat, is possible [4,14,15]. Some authors associated maras with tree cover, probably because trees offer them several resources, such as food and protection against climate conditions and predation. Tree cover can represent several hierarchical levels of resource aggregation distinguished by the mara at its finest scale of perception, as suggested by the model using plant biological forms and floristic composition [43,44], but that could represent a trade-off between the resources offered by the woodland and the increase in the predation risk in this more complex environment [45]. Our results highlighted the importance of mesquite trees in this hyper-arid ecosystem, being thus consistent with findings of a recent study in a semiarid ecosystem by [17], but differing from previous studies that stress the importance of open scrubland [14,15,16].

The effects of anthropogenically perturbed areas on mara habitat use are controversial. It is known that the mara is more associated with degraded lands outside protected areas than with the protected areas, because livestock generates open lands [14,17]. Furthermore, maras use roads and trails, and the continuing expansion of human activities on natural environments probably forces maras to use open environments created by humans [16]. However, other authors suggest human-induced habitat degradation (e.g., overgrazing) and poaching to be major processes negatively affecting mara populations [46,47]. Our study is the first report on the effects of tourism activities on the mara. Maras used more areas near the tourist circuit than far away from it. It is known that the mara uses roads and trails as corridors for moving between areas used in other regions [16]. Therefore, our results provided evidence that in a protected area, where tourism activity is intense, maras select areas near the tourist circuit. Based on these findings and the behavior of other herbivors [48,49,50], maras could perceive the areas with high tourist activity, such as the tourist circuit, as safer, possibly as a place safe from predators, and this could be triggered by the effect of fear of humans [51], although we still need many studies to demonstrate the causes of these changes in mara’s use of the environment.

There are several factors involved in the decline of the mara: habitat loss by overgrazing [15], hunting pressure [3,52], infectious diseases [15], and dietary overlap with European hares [10]. Within this framework, our study found that maras do not avoid areas with touristic activity.

Maras used areas near the touristic circuit, probably because they do not perceive humans as a threat, at least in the protected area with the current tourism activity. Our finding is relevant because this protected area is a World Heritage site with more than 95,000 visitors annually [28,29]. However, we suspect tourism activity will increase once the Central bi-oceanic corridor construction is finished. This linear infrastructure is an important road (with an extension of 2500 km) that will connect two ports: Porto Alegre (Brazil) with Coquimbo (Chile). It will run across the entire central area of Argentina, including the southern sector of the Ischigualasto Provincial Park [53]. This will allow a greater tourist arrival in the protected area. Although currently the tourist activity within the park is well managed, below its ecological tourist carrying capacity, we suppose that constant monitoring of tourist activities and its effects on fauna is necessary.

## 5. Conclusions

We found complex interactions of abiotic and biotic variables with tourist activity. Maras selected higher *Prosopis* cover when present near a touristic circuit, but not when they were away from the touristic circuit. Maras also selected areas with low bare soil cover in the studied area, differing from other studies that found maras selecting places with high bare soil cover. Managers thus face the challenge of finding strategies that may promote the coexistence of wildlife and human activities in a diverse world where ecological interactions are complex.

## Figures and Tables

**Table 1:** The best models of habitat use of mara for each biological study in the Ischigualasto Provincial Park.

Biological Levels	K	AICc	ΔAICc	D^2^
Plant Community				
Pebble Cover + Bare Soil	4	486.82	0	15.42%
Null Model	2	497.00	10.18	0%
Plant Strata				
Pebble Cover + Bare Soil	4	486.82	0	15.42%
Null Model	2	497.00	10.18	0%
Biological Form				
Tourism Activity + Pebble Cover + Bare Soil +Trees + Tourism Activity × Pebble Cover + Tourism Activity × Trees	8	476.80	0	31.31%
Null Model	2	497.00	10.18	0%
Floristic Composition				
Tourism Activity + Pebble Cover + *Prosopis* + Bare Soil + Tourism Activity × Pebble Cover + Tourism Activity × *Prosopis*	8	476.80	0	31.31%
Null Model	2	497.00	10.18	0%

Note: K, number of parameters in each model; AICc, corrected Akaike information criterion; ΔAICc, difference between the model with the lowest AICc value and each candidate model from best to worst; D^2^, deviance explained. In models, + signs addition, and × signs indicate interaction.
